# Immunotherapy for Pediatric Brain Tumors

**DOI:** 10.3390/brainsci7100137

**Published:** 2017-10-21

**Authors:** Elias J. Sayour, Duane A. Mitchell

**Affiliations:** University of Florida Brain Tumor Immunotherapy Program, Preston A. Wells, Jr. Center for Brain Tumor Therapy, Lilian S. Wells Department of Neurosurgery, 1149 South Newell Drive, McKnight Brain Institute, University of Florida, Gainesville, FL 32611, USA; duane.mitchell@neurosurgery.ufl.edu

**Keywords:** immunotherapy, pediatric brain tumors, vaccines

## Abstract

Malignant brain tumors are the most common cause of solid cancer death in children. New targeted therapies are vital to improve treatment outcomes, but must be developed to enable trafficking across the blood brain barrier (BBB). Since activated T cells cross the BBB, cancer immunotherapy can be harnessed to unlock the cytotoxic potential of the immune system. However, standard of care treatments (i.e., chemotherapy and radiation) applied concomitant to pediatric brain tumor immunotherapy may abrogate induction of immunotherapeutic responses. This review will discuss the development of immunotherapies within this paradigm using emerging approaches being investigated in phase I/II trials in children with refractory brain tumors, including checkpoint inhibitors, vaccine immunotherapy, and adoptive cell therapy.

## 1. Introduction

Malignant brain tumors are the most common solid tumor in children [1,2,3]. Therapy is generally confined to maximal surgical resection, followed by adjuvant radiotherapy, and combination chemotherapy [4,5,6]. Despite improvements in outcomes, survivors often suffer toxicities from off-target effects, including incapacitating neurological deficits and cognitive decline [7]. Due to the debilitating side effects of surgery, radiation, and chemotherapy, new therapies are vital [7]. However, pediatric brain cancers are particularly challenging due to the blood brain barrier (BBB) and a lack of approved novel agents [8]. Most chemotherapeutic agents have limited penetration of the BBB [9,10,11,12,13]. For drugs that do penetrate (i.e., temozolomide), systemic levels typically dwarf therapeutic levels in the central nervous system (CNS), which compounds their secondary side effects [9,10,11,12,13]. New agents, such as tyrosine kinase inhibitors, may have limited penetration of the capillary tight junctions formed by endothelial cells of the BBB [14,15]. This endothelial barrier is reinforced by an epithelial barrier formed by the choroid plexus at the interface between the blood and cerebrospinal fluid [16]. Hence, translocation across these barriers is difficult to achieve from pharmacologic therapies, and direct intratumoral injection is encumbered by need for surgical access and indwelling catheters that are prone to increased edema, bleeding, and infection [14,17]. Given these limitations, new agents are necessary for specific targeting of brain tumor cells without the bystander damage synonymous with traditional chemotherapeutic agents.

## 2. Immunotherapy and Chemoradiation

Cancer immunotherapy is a burgeoning field of targeted therapy that harnesses the cytotoxic potential of the immune system against cancer cells. T cells are the effector arm responsible for flushing out foreign pathogens that pretend to be ‘self’. Viral infections typically hijack cell machinery, but major histocompatibility complex (MHC) class presented epitopes allow T cells to recognize and reject viral antigens on infected host cells [18]. Similarly, the preponderance of cancer cells are composed of normal antigens, but can be riddled with neoeptiopes sensitive to immunotherapeutic targeting [19,20,21]. Unlike traditional pharmacologic agents that require increased titration for full effect (often with dose limiting toxicities that preclude rampant dose escalation), immunotherapy is a biologic agent that may not necessitate the same dose-dependent increases for maximal impact [22]. These biologic agents require a catalyst to activate/reactivate the endogenous host response responsible for unlocking adaptive immunity and perpetuating self-sustaining immunologic T cell memory against malignant brain tumors [23]. Despite the BBB, the concept of the CNS being ‘immune-privileged’ is no longer adequate [24,25,26,27,28]. Recently, lymphatic drainage networks have been described within the CNS that communicate with deep cervical draining lymph nodes [24,25,26,27,28]. Activated T cells can be primed against tumor-specific antigens and traverse the BBB through adhesion markers (i.e., VLA-4), allowing them to penetrate the tumor microenvironment and induce their effector functions against pediatric cancer cells [24,25,26,27,28].

For children with malignant brain tumors, immunotherapy may be employed in conjunction with standard of care therapy. Since standard treatment typically involves surgery, radiation, and chemotherapy, the timing of immunotherapy must be reconciled to mitigate against the detrimental effects associated with these traditional modalities. Radiation may promote angiogenesis/wound healing and confound Th1 type responses by decreasing CD8+ dendritic cells [29,30]. However, radiation can synergize with immunotherapy through the release of damage associated molecular patterns (DAMPs) and tumor antigens that can be processed by endogenous antigen presenting cells (APCs) for presentation to T cells in local draining lymph nodes [31]. Chemotherapy, like radiation, can have both positive and negative effects when used in conjunction with immunotherapy [31]. Chemotherapy can be directly toxic to activated T cells, but may eliminate regulatory populations (i.e., regulatory T cells, myeloid derived suppressor cells) that compete for essential cytokines [32,33,34]. Removal of these cell compartments acting as cytokine sinks allows immunotherapeutic agents to induce their effector responses without competition [32,33,34]. To engender T cell or NK cell survival, adoptive cellular therapy is best utilized after radiation and chemotherapy; however, vaccine immunotherapy may synergize in conjunction with these treatment modalities [35,36]. Vaccines prime innate immunity for polarization into an adaptive T cell response and may synergize with the inflammatory milieu induced by radiation and chemotherapy [31,35,36]. Preclinical and clinical investigations have shown that vaccination through repeated cycles of chemotherapy enhances antigen specific T cell immunity [36,37,38]. Based on these data, future immunotherapy trials for children with malignant brain tumors can be developed in conjunction with standard of care chemoradiation. Vaccines may be optimal soon after surgical resection for continuation through cycles of adjuvant chemotherapy. Adoptive cellular therapy, if also utilized, can follow after completion of chemotherapy with continued vaccination cycles to maintain immunologic memory.

## 3. Antigen Selection

To incite an immune response against pediatric brain tumors, appropriate tumor antigens have to be selected and targeted. While nonsynonymous mutations that generate neoantigens are optimal targets (since they are not expressed on normal tissue), these are likely personalized epitopes that differ between patients, and are less prevalent in tumors with low mutational burdens (i.e., childhood CNS malignancies) [25]. Alternative targets include differentiation antigens or overexpressed normal antigens, but immunologic targeting of epitopes expressed on normal tissue may elicit autoimmunity [39]. Although most pediatric brain tumors have scant mutations at the genomic level, they are characterized by a plethora of epigenetic dysregulatory mechanisms, which may lead to the re-expression of developmental fetal antigens in malignancies, such as medulloblastoma and primitive neural ectodermal tumors (PNETs) [40,41,42]. In these malignancies, developmental antigens may be identifiable and targetable as tumor specific epitopes not expressed on mature normal tissue. Due to clustering of many pediatric brain tumors into distinct subgroups (based on expression profiles, medulloblastomas can be clustered into four distinct subgroups [43], and recently have been further subdivided into seven subgroups [44], while ependymomas can be stratified based on supratentorial or posterior fossa location [45]), there may be conserved targets unique to each subtype. Interestingly, diffuse intrinsic pontine gliomas (DIPGs) typically have a conserved mutation in histone 3.3 or 3.1 (K27M) [46]. This mutation may allow targeted immunotherapy in a surgically unresectable tumor against novel epitopes spanning the region of the K27M mutation [6,47,48]. Currently, H3.3K27M specific cancer vaccines are being evaluated in early phase studies for children with H3.3K27M positive DIPGs (clinicaltrials.gov: NCT02960230). 

## 4. Immunotherapeutic Strategies

The immune system is one of the prime defense mechanisms against cancer cells, and works to actively survey and remove cells that are undergoing oncologic transformation [49,50]. Endogenous immunity is responsible for eradicating infectious etiologies and removing dysregulated pre-cancerous cells, but can also be remodeled (by malignancies) into an immunoregulatory state [51,52,53,54,55] When a critical mass of cancer cells develops, the immune system mounts a response, but consequently, selects immune resistant tumor cells to re-populate the mass [49,50]. As malignancies gain mutations they evolve to resist the immune system, while actively subverting it from inducing anti-tumor activity [56,57]. This new tumor microenvironment becomes comprised of immunoregulatory populations, including FoxP3+ regulatory T cells, tumor associated macrophages, and myeloid derived suppressor cells, which actively work to suppress effector T cells from mediating their cytolytic functions [58,59,60]. Immunotherapy can be employed to effectively reprogram the host immune response via immune checkpoint blockade, cancer vaccines, and adoptive cell therapy. 

Immune checkpoint blockade antagonizes inhibitory receptor/ligand interactions (i.e., programmed death-ligand 1 (PD-L1) on APCs and tumor cells/programmed death-1 (PD-1) receptor on T cells) designed to inhibit activated T cells [61]. Blocking these inhibitory axes with monoclonal antibodies (mAbs) allows activated T cells to ‘stay on’ and mediate their anti-tumor effector functions [61]. Checkpoint inhibitors have shown remarkable results against adult cancers (i.e., skin and lung), but pediatric malignancies are distinct [39,62]. Unlike melanoma and lung cancer, which are characterized by high mutational burdens typically acquired via environmental mutagens (i.e., ultraviolet rays predisposing skin cancer and cigarette smoke predisposing lung cancer), pediatric cancers have a low mutational burden [21,61,62]. Responsiveness of melanoma to immune checkpoint blockade has been strongly associated with mutational burdens from non-synonymous changes and intratumoral enrichment of PD-1+ CD8+ T cells [21,54,61,63,64,65,66]. Although PD-1 can serve as an exhaustion marker, it is expressed on activated T cells, which are typically stymied by the regulatory microenvironment endemic to refractory solid tumors [65]. Checkpoint inhibitors unlock these activated T cells allowing them to exert their effector functions against neoantigen tumor epitopes [21,65]. Since pediatric brain tumors have low mutational burdens, they may be devoid of these endogenous PD-1+ T cells. Moreover, immune checkpoint mAbs are not expected to cross the BBB; however, in the presence of chemotherapy and radiation, this barrier may be perturbed, allowing these molecules to translocate [25,67,68]. Clinical trials are currently underway in children with high grade gliomas (HGGs) and DIPGs (phase I, clinicaltrials.gov: NCT02359565) to determine the utility of these agents against pediatric CNS malignancies.

Since childhood brain cancer is considered as a rare disease population, the development of novel therapies in clinical trials that can be studied in large data sets is necessary. While checkpoint inhibitors (i.e., PD-1 and PD-L1 mAbs) are promising candidates that can be leveraged for all patients, they appear to require tumors with high mutational burdens and PD-1+ intratumoral T cells (or high expressing PD-L1+ tumors) [61,62]. CD47, an immune evasion marker on the surface of many solid cancers, may represent a more relevant tumor immunosuppressive target [69]. CD47 is overexpressed by malignant cells to evade the innate immune response and may be particularly enriched in pediatric brain tumors including medulloblastoma, acute teratoid rhabdoid tumor, pediatric glioblastoma, and DIPG [69]. By antagonizing CD47 (which binds to activating signal regulatory protein-alpha (SIRPalpha) on myeloid cells) with a humanized mAb, pre-clinical investigations have demonstrated that macrophages can be unlocked to phagocytize tumor cells in several pediatric brain tumor models [69]. In addition to CD47 targeting, tumor immunosuppression can be overcome through targeting indoleamine 2,3-dioxygenase (IDO) [70,71]. IDO is an enzyme enriched in malignant brain tumors and functions to deplete tryptophan, an essential amino acid, from effector T cells [70,71]. Tryptophan is critical for DC maturation and for maintaining effector T cell survival/longevity [70,71]. Tumors that utilize IDO deplete tryptophan from T cells forming kynurenines, which predispose an exhausted and regulatory T cell phenotype [70,71]. IDO blockers have been developed with promising pre-clinical results in murine brain tumor models and are currently being evaluated in children in early phase studies (phase I, clinical trials.gov: NCT02502708) [55,59].

While several strategies exist for overcoming tumor-mediated immunosuppression, in the absence of robust preexisting endogenous immunity, targeting immunosuppression may be insufficient to induce meaningful anti-tumor responses. Alternatively, strategies have been employed to activate immunologic responses de novo against pediatric brain tumors. These include cancer vaccines, oncolytic viral therapy, and chimeric antigen receptor modified T cells. Cancer vaccines seek to deliver tumor antigens to APCs allowing them to process and present these epitopes on the surface of their MHC class I and II molecules for presentation to T cells [72]. Glioma associated peptide vaccines with poly-I/C have been investigated in HLA-A2 positive patients with DIPG and HGGs [73]. These peptides (commonly expressed in childhood gliomas) included EphA2, interleukin-13 receptor alpha, and survivin [73]. The peptides were emulsified in montanide-ISA-51 and given in conjunction with poly I/C (every three weeks x 8 cycles followed by booster administrations every six weeks) [73]. Thirteen of twenty-one children treated with these peptide vaccines had an immune response to the antigens [73]. Although these results are promising, HLA-A2 restricted vaccines constrain eligibility to patients that express this haplotype. Alternatively, DNA or RNA based vaccines bypass HLA haplotype restriction, allowing an individual’s cell machinery to transcribe/translate nucleic acids into proteins for personalized processing [74,75,76,77]. Our group has employed the use of RNA loaded ex vivo activated autologous dendritic cells from patients with medulloblastoma [78]. DCs are matured ex vivo from peripheral blood mononuclear cells (obtained via leukapheresis) and activated in the presence of inflammatory cytokines [78]. These cells are loaded with a personalized cohort of total tumor messenger RNA amplified from a personalized cDNA library representing a tumor specific transcriptome [78]. Autologous DCs are cultured with T cells (also obtained via leukapheresis) to activate them before both products (RNA-loaded DCs and ex vivo activated T cells) are re-administered to patients [78]. In patients with medulloblastoma and primitive neural ectodermal tumors, investigations are currently underway in phase I and II studies (clinicaltrials.gov NCT01326104) [78].

Like vaccines, oncolytic viral therapy intends to induce endogenous immune responses [79,80,81,82,83]. Oncolytic viruses are attenuated to preferentially propagate in tumor cells (which lack normal cellular defense mechanisms) until they are recognized by the immune system for rejection [79,80,81,82,83]. As these infected cancer cells lyse, a broader immune response is engendered more globally against shed tumor antigens, in a process known as epitope spreading [79,80,81,82,83,84]. Oncolytic viral therapy has been employed in several cancers with promising results and is currently being evaluated in phase I studies in children with recurrent supratentorial tumors (using herpes simplex virus; clinicaltrials.gov: NCT02457845) [85].

To mediate tumor regression, cancer vaccines and oncolytic viral therapy stimulate innate immune responses for polarization of an antigen specific T cell response. For these strategies to be successful, appropriate orchestration of an innate stimulus, APC activation/trafficking, and presentation to T cells must occur [72]. Subsequently, T cells must penetrate the notoriously immunosuppressive microenvironment characteristic of many CNS malignancies, and induce their effector functions against tumor cells that can downregulate MHC class I, which is requisite for T cell recognition [86]. To bypass this hurdle, engineered chimeric antigen receptor (CAR) modified T cells can be utilized to target surface antigens [87,88,89,90,91,92]. Since they recognize and reject surface antigens (i.e., HER2), CAR T cells overcome MHC downregulation by tumor cells [87,88,89,90,91,92]. Recently, CAR T cells have been shown to mediate significant anti-tumor activity against adult glioblastoma by targeting HER2 or IL13Ra2, which can be employed against pediatric high-grade gliomas (phase I, clinicaltrials.gov: NCT02208362) [93,94]. CAR T cells embed the specificity of an antibody with the cytotoxicity of a T cell, but remain limited by concerns of off-target effects [87,88,89,90,91,92]. CAR T cell targeting of normal antigens in the brain may lead to CNS inflammation, neurotoxicity, and risks for an increased intracranial pressure and herniation. Future development of CAR T cell therapies that activate only after binding to multiple tumor surface antigens may limit these off-target effects [95].

Other types of adoptive cell therapies for pediatric brain tumors include the transplantation of autologous/allogeneic tumor-specific cytotoxic T lymphocytes and natural killer (NK) cells. In children with posterior fossa tumors, autologous NK cells are being be expanded ex vivo before forth ventricular infusion (via ommaya reservoir) (phase I, clinicaltrials.gov: NCT02271711). Allogeneic T and NK cells are also being administered after non-myeloablative haploidentical transplant (reduced intensity conditioning regimens) to enable graft versus tumor killing against high risk solid tumors (i.e., pediatric brain tumors) (phase II, clinicaltrials.gov: NCT01804634 and NCT02100891). While these early phase studies are important to better understand the optimal dose, safety, and efficacy of these investigational agents, combinatorial approaches are likely requisite to fully exploit the benefits of adoptive cell therapy. By antagonizing tumor induced immunosuppression and activating endogenous immunity (via vaccine/viral immunotherapy and adoptive T cell therapy), combination immunotherapy promises to synergize for maximal therapeutic benefit.

## 5. Conclusions

While aggressive surgical resection, radiation, and chemotherapy have improved the cure rates for many pediatric patients with malignant brain tumors, these cancers remain the most common cause of solid cancer death in children [96]. New targeted therapies are vital to improve treatment outcomes, but must be developed to enable trafficking across the BBB. Cancer immunotherapy is a promising treatment modality with encouraging results in melanoma, leukemia/lymphoma, and lung cancer [62,91,97]. Although immunotherapy has shown promise against peripheral cancers, it remains challenged by the perceived lack of immunogenic/targetable antigens in pediatric brain tumors. However, given the emergence of monoclonal antibodies targeting checkpoint molecules and ‘don’t eat me’ signals (i.e., CD47), we can now unlock innate and adaptive immunity in a non-specific manner. Although the BBB remains a significant hurdle, activated T cells can cross this barricade. Tumor specific T cells can be activated de novo through cancer vaccines, oncolytic viral therapy, and ex vivo engineered cell therapies. As new targets are uncovered from subgroup speciation of pediatric brain tumors, novel therapies are expected to follow with improved outcomes.
